# Supplementary material for: A Machine Learning Model to Estimate Toxicokinetic Half-Lives of Per- and Polyfluoro-Alkyl Substances (PFAS) in Multiple Species
Source: Toxics. 2023 Jan 20;11(2):98. doi: 10.3390/toxics11020098 (PMC9962572; doi:10.3390/toxics11020098)
Supplement: Supplementary file 1 [file toxics-11-00098-s001.zip › toxics-2125126-supplementar2.9 update/S1_Dawson et al._ML PFAS_HL_101322.pdf]

A Machine Learning Model to Estimate Toxicokinetic Half-lives of Per- and Polyfluoro-alkyl Substances (PFAS) in Multiple Species

Daniel E. Dawson[1], Christopher S. Lau[2], Prachi Pradeep[1,3], Risa R. Sayre[1,3], Richard S. Judson[1], Rogelio Tornero-Velez[1], and John F. Wambaugh[1]

[1]U.S. Environmental Protection Agency, Office of Research and Development, Center for Computational Toxicology and Exposure, 109 T.W. Alexander Drive, Research Triangle Park, NC 27709

[2]U.S. Environmental Protection Agency, Office of Research and Development, Center for Public Health and Environmental Assessment, 109 T.W. Alexander Drive, Research Triangle Park, NC 27709

[3]Oak Ridge Institutes for Science and Education, Oak Ridge, TN 37830

Supplemental Information S1: Methods

S1.1 Half-Life Dataset assembly

S1.1.1 Dependent variable

We modeled in vivo serum t1/2 data for 11 PFAS chemicals using published experimentally collected data from four species. Chemicals included Perfluorooctanoic acid (PFOA), Perfluorooctanesulfonic acid (PFOS), Perfluorobutanesulfonic acid (PFBS), Perfluoroheptanesulfonic acid (PFHpS), Perfluorohexanesulfonic (PFHxS) acid, Perfluorobutanoic acid (PFBA), Perfluoropentanesulfonic acid (PFPeS), Perfluorohexanoic acid (PFHxA), Perfluorodecanoic Acid (PFDA), Perflouro-2-methyl-3-oxahexanoic acid (GenX) and Perfluoro(2-((6-chlorohexyl)oxy)ethanesulfonic acid (F-53B). The latter two chemicals are commercial formulations and, respectively, consist of the desalted chemical for Genx and the major component of what is usually a racemic compound for F-53B. Species include humans, monkey (presumably Macaca fascicularis, the cynomolgus monkey), mouse (presumably Mus musculus), and rat (presumably Rattus rattus). Data from both sexes of each species were also included, as available. See the Supplemental Information (S3.1) for all raw values extracted from the literature.

Data presented in the literature consisted of measured and calculated mean $t_{1/2}$ values per species, sex, and chemical that were usually accompanied by measures of variance (standard deviation, standard error, or 95% confidence interval). Some data sources presented $t_{1/2}$ as single point values. Some sources for non-human studies also considered the effects of dose concentration on toxicokinetic parameters, dosing method (oral gavage (Oral) versus intravenous

(IV)) and dose frequency (single versus multiple). While some studies used non-compartmental models and, thus, presented a single parameter for $t_{1/2}$, multiple studies used compartmental toxicokinetic models and presented both initial (α) and longer-term (β, γ) phases of half-life. In addition, while some chemicals/species/sex/dosing methods were represented by single values or value ranges from single papers, others had values reported from multiple sources.

To generate a single value of $t_{1/2}$ value per chemical, species, sex, and dosing method for our training set, we processed the available data in the following way. First, if a single literature value was available per chemical/species/sex/dosing method, that value was used. If multiple phases were presented (that is, α,β), only the last phase was selected. If only a single value was available for both sexes of species/dosing method, that value was assumed for both sexes/dosing method. If only replicate values were available per chemical/species/sex/dosing method without measures of variance, a mean of the available values was used. When data from multiple sources were available with measures of variance, we used a Monte Carlo approach to generate random samples from within individual ranges of presented values using standard errors (SE) as the bounds of the range. If standard deviations were presented, they were converted to SE and were divided by the square root of the sample size noted in the publication (or 3 if not noted, the minimum required for a standard error, and the size often used in vivo toxicokinetic studies). If 95% confidence intervals were presented, bounds were divided by 1.96. Some chemical/species/sex/dosing method combinations had a mixture of sources that included some with both means and measures of variance and some that had single point estimates. In this situation, the mean standard deviation of the sources with presented standard deviations was calculated. Then, it was assigned as a standard error to each single estimate by dividing it by the sample size for the point estimate listed in the text of the source publication. In the case that a

source contributed a point estimate from a single animal, it was assigned a standard error of 0. Distributions were generated by randomly sampling N animals (N = the sample size used in generating the source estimate) from within the SE bounds assigned to each source, storing these samples in a vector, and then repeating this process 100 times. Thus, each contributing study was proportionately represented in the complete vector of sampled values. Lastly, we fit a distribution to all combined in silico generated data points and used the mean of this distribution as the $t_{1/2}$ value in our training set for the corresponding chemical/species/sex/dosing method. Distributions were fit using the R package fitdistrplus[1], and an appropriate distribution (between the normal, lognormal, gamma, and exponential) was chosen based on the lowest AIC score.

The distribution of available datapoints was inconsistent per species and sex, resulting in a total of 91 datapoints used in model construction. Of these, 50 were distinct measures by species and sex. See the Supplemental Information (S2.2) for the compiled processed dataset used for QSPR model construction.

S1.1.2 Independent variables

Descriptors	Total Considered	Examined with Recursive Feature Elimination	Parsimonious Model
Physiological	21	21	4
Critical Micellular Concentration	2	0	0 (could not be predicted)
Endogenous Ligand Similarity	67	67	3
Serum Albumin Binding	3	3	1
Liver fatty acid binding protein	2	2	1
Physico-chemical	22	22	5
Categorical	2	0	2
Total	119	115	16

Table S1.1 Counts of Descriptors Considered

An important challenge in using machine learning techniques is in assembling a suitable set of descriptors. In the case of PFAS, differences between species and sex that complicate model estimation are likely driven by a mix of physiological and chemical differences. First, PFAS excretion from blood is largely accomplished through urine via the kidneys.[2, 3] Furthermore, PFAS chemicals may be re-absorbed in the kidney by receptors intended to re-uptake fatty acids, a process influenced by sex in some species.[3, 4] For example, hexanoic acid ($C_6H_{12}O_2$) is a fatty acid naturally found in animal fats that is analogous in structure to Perfluorohexanoic acid (PFHxA) ($C_6HF_{11}O_2$). The excretion and re-uptake of both compounds in the kidney might be accomplished through similar receptor mediated transporters [5]. If PFAS activate similar metabolic pathways of endogenous compounds more generally, similarity to endogenous compounds may be a predictor

of $t_{½}$. Lastly, PFAS have various chemical properties and structures depending upon their intended use. For example, some tend to form micelles, be more water soluble, or contain specific chemical structures that promote or retard metabolism. Therefore, both chemical properties and particular structures likely influence $t_{½}$.

With this information in mind, we assembled a set of chemical and physiological descriptors as potential predictors of $t_{½}$. These data included:

Physio-chemical characteristics: Serving as the basis of our descriptor dataset, a suite of 17 physio-chemical characteristics predicted by the OPERA modeling platform [6] were associated with each PFAS chemical. OPERA models themselves have defined applicability domains based on both the local and global neighborhood measures. For our training dataset, we only included OPERA models as predictors if at least half of the training chemicals in our dataset fell within the global domain of the model and had nearest neighbor scores of at least 50% (that is, within local domain). We also included average molecular mass as a potential predictor, available from the USEPA CompTox Dashboard[7].

Kidney characteristics. As the kidney is a primary site of PFAS metabolism[8], and metabolism may be influenced by physical aspects of the kidney, a suite of kidney structure characteristics (for example kidney weight, number of nephrons, glomerular surface area) was assembled from Oliver [9]. For species missing from Oliver [9] (Monkey), data for the four primary descriptors (body weight, kidney weight, proximal tubule length, and proximal tubule diameter) used in calculating other descriptors were interpolated via linear regression of Log10-transformed kidney weight. [10, 11] All regressions were significant ($p<0.05$) and had $R^2 \geq 0.67$).

Endogenous Chemicals. The similarity of exogenous and endogenous chemicals may influence how exogenous chemicals are metabolized in the body,[12] and several PFAS chemical have similar non-fluorinated endogenous analogs. To incorporate this information into a predictor dataset, we identified a set of 894 endogenous compounds (include method here). To assess the similarity between PFAS compounds and endogenous compounds, we calculated two sets of molecular descriptors, including PubChem and Morgan fingerprints. The PubChem method simply constructs a fingerprint based on the presence of absence of a list of molecular characteristics.[13] The latter method is called a “circular” molecular descriptor, as it describes molecular structures by way of circular atom neighborhoods.[14] We then calculated similarity scores between this set and each included PFAS compound using the Tanimoto method (that is, Jaccard similarity).[15] The Tanimoto scores each of these chemicals for each PFAS chemical was then assembled. From here, we reasoned that molecules with the highest or lowest similarities with PFAS chemicals might be useful as predictors of toxicokinetic properties. Therefore, the endogenous chemicals with the highest and lowest similarity score with each PFAS chemical were identified. Then, all of the unique chemicals in this “high-low” set were assembled, along with their similarity scores. Finally, because xenobiotics generally have to be very similar to endogenous compounds to activate molecular receptors (citation), we discretized similarity values at a threshold value >=0.9 being either similar (1) or dissimilar (0). We also considered using the maximum similarity between any endogenous chemical in the set as a predictor. However, sets of endogenous chemicals are likely to change in the future, and pairwise comparisons between many chemicals are computationally expensive. As this would made applications to new chemicals challenging, we decided to not include this as predictor.

Protein Binding. Perfluorinated chemicals bind to specific proteins in the liver and to albumin in serum, which likely influences metabolism rates[8]. To account for this, experimentally derived serum-albumin binding rate constants ($Ka(M^{-1})$) [4] and binding rate disassociation constants to the fatty acid binding protein (L-FABP) [16] were added for a subset of available PFAS chemicals.

Ether bond. Some perfluorinated chemicals have been designed to include an ether bond in order to facilitate more rapid metabolism. To account for this, a binary descriptor was included denoting the inclusion of an ether bond along the carbon backbone.

Non-included Predictors. Two predictor types that were considered for inclusion but ultimately were not included were carbon chain length and critical micelle concentration (CMC). Carbon chain length is a commonly considered structural characteristic that influences chemical properties.[17] However, the definition of carbon chain length is not obvious or consistent for non-straight chained PFAS chemicals, and the implications of specific definitions of this characteristic is beyond the scope of this paper. For the CMC, PFAS chemicals form micelles above a concentration, referred to as the CMC.[17, 18] As the nature of their metabolism may change as hydro- and lipophilic moieties alter past the CMC[18], it may be a useful, important predictor of serum half-life. However, a current model of CMC[18] utilizes a descriptor produced via a proprietary software (Dragon), making this predictor not readily extendable to novel predictions. The exclusion of these two descriptors here is compensated by preliminary work showing that both were strongly correlated with other considered predictors.

S1.1.3 Descriptor Assembly

When all predictors were assembled, the missing values of descriptors for certain species/chemical combinations were interpolated in two steps. First, if values for chemicals were

missing for a particular species, then the average of the values of available species was assigned to the missing species. If no values were available for any species for a particular chemical, then the average value for all available values of that chemical was used. The result was a dataset with 91 records and 98 potential predictors.

Machine learning techniques are prone to overfitting, particularly when there are more predictors than dependent data points. This problem is made worse when predictor variables are strongly correlated. To reduce these issues, we first identified and eliminated low variance descriptors, defined as descriptors with standard deviation/mean < 0.05. Next, we calculated Spearman correlation coefficients between each pair of predictor variables and identified all pairs of predictors that were strongly correlated (Spearman's ρ=0.9). Then, we used the "findCorrelation" function of the caret[19] package of program R to evaluate each pair of highly correlated descriptors and eliminate the more globally correlated descriptors. The result of this process was a set of 14 numeric descriptors, which were then mean-centered and scaled by standard deviation. Finally, we added two categorical descriptors, including sex (male, female), and dosing type as indicated in the literature source documentation (intravenous, oral, other (epidemiological, via metabolite extrapolation)). As the final models were intended to be applied to multiple species, the species themselves were not included as descriptors. In addition, kidney types of the species in the model only included two (multirenculated and unipapillary) out of multiple kidney types. Therefore, kidney type was also not included as predictor.

See the Supplemental Information (S3.3) for the complete training set used for QSPR model construction. Note that predictor values were zero-centered and scaled for model construction.

S1.2 Volume of Distribution (Vd) Modeling

S1.2.1 Dataset Assembly

In parallel with the construction of a QSPR model for $t_{1/2}$, we used the same approach to attempt to develop a predictive model for the toxicokinetic volume of distribution (Vd), in units of l/kg body weight. This is a theoretical parameter representing the distribution of a chemical in the body of an organism by relating the concentration apparent in the plasma with the total amount of PFAS present in the body. As Vd cannot be measured directly, it is estimated from fitting models to empirical data. A dataset of literature-derived values of Vd was compiled, starting from Pizzuro et al. (2019) Table 2,20 which compiled 38 observations spanning five PFAS chemicals, four species, and both sexes from various literature sources.21-30 To these, we added 24 calculated Vd observations in rats for three chemicals across a range of doses and routes from Huang et al. (2019).31 Huang et al. (2019) fit their time-series data with a two compartment model parameterized in terms of a primary compartment (V1), α and β rates, and an overall elimination rate (kelim), and so we used Vd,ss = V1*(1+k12/k21), where k12 and k21 are the rates between the first and second compartments and

$$\alpha = \frac{1}{2} * \left(k_{elim} + k_{12} + k_{21} + \sqrt{(k_{elim} + k_{12} + k_{21})^2 - 4 * k_{elim} * k_{21}}\right)$$

$$\beta = \frac{1}{2} * \left(k_{elim} + k_{12} + k_{21} - \sqrt{(k_{elim} + k_{12} + k_{21})^2 - 4 * k_{elim} * k_{21}}\right)$$

as given by O' Flaherty [32]. Calculations for Huang are included in the Supplemental Information (S2.6). Further values for *Vd* were collected from [33-36]. The total data set includes 128 values for

Vd from eight PFAS chemicals across four species. See Supplemental Information S2.5 for all Vd values used in model construction.

S1.2.2 Model construction

Although the available descriptors were chosen to allow the modeling of half-life, all the same descriptors (including kidney structure) were made available to the machine learning approaches for Vd. As with half-life, feature selection was used to reduce the number of possible predictors to be analyzed. We used the random forest method.

S1.2.3 Modelling Results and Discussion

The accuracy of the models with three, four, or five bins ranged from 56% to 37%. A two-bin model had a 70% accuracy, but the central tendencies of the two bins were distinct from the medians of any chemical or species. Thus, the two-bin model seemed to capture the uncertainty in the data more than any chemical or species differences.

The volume of distribution depends on the chemical partitioning into various tissues[32], which is a function of the tissue composition (water, lipids, proteins, membranes)[37] and tissue volumes .[38] Interspecies differences are essentially driven by relative differences in tissue volumes and, to a lesser extent, composition. Differences between chemicals are expected to be driven by differences of affinity for the tissue constituents. In comparison to $t_{1/2}$, the compiled values for Vd varied relatively little, with median Vd values ranging across chemicals from 0.139 to 0.368 L/kg, and across species from 0.194 to 0.254 L/kg. Thus, the present failure to build more compelling models for predicting inter-chemical and -species differences is at least partially a function of the

lack of variability among the data relative to the strong uncertainty. In addition, for only PFOS in the rat, Vd ranged widely from 0.0886 to 7.01 L/kg. This broad uncertainty likely confounded attempts to build a better predictive model. Therefore, until something more compelling is developed, possibly through additional data generation and curation, we believe that the data set median of 0.202 L/kg bodyweight is a reasonable default value for Vd.

References

1. Delignette-Muller, M. L.; Dutang, C., fitdistrplus: An R Package for Fitting Distributions. *Journal of Statistical Software* **2015,** *64*, (4), 1-34.

2. Jian, J. M.; Chen, D.; Han, F. J.; Guo, Y.; Zeng, L. X.; Lu, X. W.; Wang, F., A short review on human exposure to and tissue distribution of per- and polyfluoroalkyl substances (PFASs). *Sci. Total Environ.* **2018,** *636*, 1058-1069.

3. DeWitt, J. C., *Toxicological effects of Perfluoroalkyl and Polyfluoroalkyl substances*. 1st ed.; Humana Press: Switzerland, 2015; p 495.

4. Han, X.; Nabb, D. L.; Russell, M. H.; Kennedy, G. L.; Rickard, R. W., Renal Elimination of Perfluorocarboxylates (PFCAs). *Chem. Res. Toxicol.* **2012,** *25*, (1), 35-46.

5. Andersen, M. E.; Clewell, H. J.; Tan, Y.-M.; Butenhoff, J. L.; Olsen, G. W., Pharmacokinetic modeling of saturable, renal resorption of perfluoroalkylacids in monkeys—probing the determinants of long plasma half-lives. *Toxicology* **2006,** *227*, (1), 156-164.

6. Mansouri, K.; Grulke, C. M.; Judson, R. S.; Williams, A. J., OPERA models for predicting physicochemical properties and environmental fate endpoints. *J Cheminform* **2018,** *10*, (1), 10.

7. EPA, U. CompTox Chemicals Dashboard. https://comptox-prod.epa.gov/dashboard/. (01/15/2020),

8. Lau, C., Perfluorinated Compounds: An Overview. In *Toxicological Effects of Perfluoroalkyl and Polyfluoroalkyl Substances*, DeWitt, J. C., Ed. Springer-Verlag London Ltd: Godalming, 2015; pp 1-21.

9. Oliver, J., *Nephrons and Kidneys: A Quantitative Study of Developmental and Evolutionary Mammalian Renal Architectonics*. Hoeber Medical Division, Harper & Row: 1968.

10. Maurya, H.; Kumar, T.; Kumar, S., Anatomical and Physiological Similarities of Kidney in Different Experimental Animals Used for Basic Studies. *Journal of Clinical & Experimental Nephrology* **2018,** *03*, (02).

11. Mandikian, D.; Figueroa, I.; Oldendorp, A.; Rafidi, H.; Ulufatu, S.; Schweiger, M. G.; Couch, J. A.; Dybdal, N.; Joseph, S. B.; Prabhu, S.; Ferl, G. Z.; Boswell, C. A., Tissue Physiology of Cynomolgus Monkeys: Cross-Species Comparison and Implications for Translational Pharmacology. *Aaps J* **2018,** *20*, (6), 107.

12. O'Hagan, S.; Kell, D. B., Understanding the foundations of the structural similarities between marketed drugs and endogenous human metabolites. *Front Pharmacol* **2015,** *6*, 105.

13. Bolton, E. E.; Wang, Y.; Thiessen, P. A.; Bryant, S. H., PubChem: integrated platform of small molecules and biological activities. In *Annual reports in computational chemistry* Elsevier: 2008; Vol. 4, pp 217-241.

14. Morgan, H. L., The generation of a unique machine description for chemical structures-a technique developed at chemical abstracts service. *The Journal of Chemical Documentation* **1965,** *5*, (2), 107-113.

15. Tanimoto, T. T., *Elementary mathematical theory of classification and prediction.* IBM: New York, 1958; p 10.

16. Zhang, L.; Ren, X. M.; Guo, L. H., Structure-based investigation on the interaction of perfluorinated compounds with human liver fatty acid binding protein. *Environ Sci Technol* **2013,** *47*, (19), 11293-301.

17. Buck, R. C.; Murphy, P. M.; Pabon, M., Chemistry, Properties, and Uses of Commercial Fluorinated Surfactants. In *Polyfluorinated Chemicals and Transformation Products*, 2012; pp 1-24.

18. Bhhatarai, B.; Gramatica, P., Prediction of aqueous solubility, vapor pressure and critical micelle concentration for aquatic partitioning of perfluorinated chemicals. *Environ Sci Technol* **2011,** *45*, (19), 8120-8.

19. Kuhn, M., Caret: Classification and regression training. R package version 6.0-85. In R Project, https://CRAN. R-project. org/package= caret: 2020.

20. Pizzurro, D. M.; Seeley, M.; Kerper, L. E.; Beck, B. D., Interspecies differences in perfluoroalkyl substances (PFAS) toxicokinetics and application to health-based criteria. *Regul Toxicol Pharmacol* **2019,** *106*, 239-250.

21. ATSDR *Toxicological Profile for 1,4 Dioxane*; Agency for Toxic Substances and Disease Registry, U.S. Department of Health and Human Services, Public Health Service: Atlanta, GA, 2012.

22. Butenhoff, J. L.; Kennedy, G. L., Jr.; Hinderliter, P. M.; Lieder, P. H.; Jung, R.; Hansen, K. J.; Gorman, G. S.; Noker, P. E.; Thomford, P. J., Pharmacokinetics of perfluorooctanoate in cynomolgus monkeys. *Toxicol Sci* **2004,** *82*, (2), 394-406.

23. Chang, S. C.; Das, K.; Ehresman, D. J.; Ellefson, M. E.; Gorman, G. S.; Hart, J. A.; Noker, P. E.; Tan, Y. M.; Lieder, P. H.; Lau, C.; Olsen, G. W.; Butenhoff, J. L., Comparative pharmacokinetics of perfluorobutyrate in rats, mice, monkeys, and humans and relevance to human exposure via drinking water. *Toxicol Sci* **2008,** *104*, (1), 40-53.

24. Chang, S. C.; Noker, P. E.; Gorman, G. S.; Gibson, S. J.; Hart, J. A.; Ehresman, D. J.; Butenhoff, J. L., Comparative pharmacokinetics of perfluorooctanesulfonate (PFOS) in rats, mice, and monkeys. *Reprod. Toxicol.* **2012,** *33*, (4), 428-440.

25. Kim, S. J.; Heo, S. H.; Lee, D. S.; Hwang, I. G.; Lee, Y. B.; Cho, H. Y., Gender differences in pharmacokinetics and tissue distribution of 3 perfluoroalkyl and polyfluoroalkyl substances in rats. *Food Chem. Toxicol.* **2016,** *97*, 243-255.

26. Kim, S. J.; Shin, H.; Lee, Y. B.; Cho, H. Y., Sex-specific risk assessment of PFHxS using a physiologically based pharmacokinetic model. *Arch. Toxicol.* **2018,** *92*, (3), 1113-1131.

27. Ohmori, K.; Kudo, N.; Katayama, K.; Kawashima, Y., Comparison of the toxicokinetics between perfluorocarboxylic acids with different carbon chain length. *Toxicology* **2003,** *184*, (2-3), 135-140.

28. Olsen, G. W.; Chang, S. C.; Noker, P. E.; Gorman, G. S.; Ehresman, D. J.; Lieder, P. H.; Butenhoff, J. L., A comparison of the pharmacokinetics of perfluorobutanesulfonate (PFBS) in rats, monkeys, and humans. *Toxicology* **2009,** *256*, (1-2), 65-74.

29. Sundstrom, M.; Chang, S. C.; Noker, P. E.; Gorman, G. S.; Hart, J. A.; Ehresman, D. J.; Bergman, A.; Butenhoff, J. L., Comparative pharmacokinetics of perfluorohexanesulfonate (PFHxS) in rats, mice, and monkeys. *Reprod. Toxicol.* **2012,** *33*, (4), 441-451.

30. Thompson, J.; Lorber, M.; Toms, L. M. L.; Kato, K.; Calafat, A. M.; Mueller, J. F., Use of simple pharmacokinetic modeling to characterize exposure of Australians to perfluorooctanoic acid and perfluorooctane sulfonic acid. *Environ. Int.* **2010,** *36*, (4), 390-397.

31. Huang, M. C.; Dzierlenga, A. L.; Robinson, V. G.; Waidyanatha, S.; DeVito, M. J.; Eifrid, M. A.; Granville, C. A.; Gibbs, S. T.; Blystone, C. R., Toxicokinetics of perfluorobutane sulfonate (PFBS), perfluorohexane-1-sulphonic acid (PFHxS), and perfluorooctane sulfonic acid (PFOS) in male and female Hsd:Sprague Dawley SD rats after intravenous and gavage administration. *Toxicol Rep* **2019,** *6*, 645-655.

32. O' Flaherty, E., J. , *Toxicants and drugs: kinetics and dynamics*. John Wiley & Sons. : 1981.

33. Dzierlenga, A. L.; Robinson, V. G.; Waidyanatha, S.; DeVito, M. J.; Eifrid, M. A.; Gibbs, S. T.; Granville, C. A.; Blystone, C. R., Toxicokinetics of perfluorohexanoic acid (PFHxA), perfluorooctanoic acid (PFOA) and perfluorodecanoic acid (PFDA) in male and female Hsd:Sprague dawley SD rats following intravenous or gavage administration. *Xenobiotica* **2020,** *50*, (6), 722-732.

34. Lau, C.; Rumpler, J.; Das, K. P.; Wood, C. R.; Schmid, J. E.; Strynar, M. J.; Wambaugh, J. F., Pharmacokinetic profile of Perfluorobutane Sulfonate and activation of hepatic nuclear receptor target genes in mice. *Toxicology* **2020,** *441*, 152522.

35. Lou, I. C.; Wambaugh, J. F.; Lau, C.; Hanson, R. G.; Lindstrom, A. B.; Strynar, M. J.; Zehr, R. D.; Setzer, R. W.; Barton, H. A., Modeling Single and Repeated Dose Pharmacokinetics of PFOA in Mice. *Toxicol Sci* **2009,** *107*, (2), 331-341.

36. Tatum-Gibbs, K.; Wambaugh, J. F.; Das, K. P.; Zehr, R. D.; Strynar, M. J.; Lindstrom, A. B.; Delinsky, A.; Lau, C., Comparative pharmacokinetics of perfluorononanoic acid in rat and mouse. *Toxicology* **2011,** *281*, (1-3), 48-55.

37. Poulin, P.; Haddad, S., Toward a New Paradigm for the Efficient In Vitro-In Vivo Extrapolation of Metabolic Clearance in Humans from Hepatocyte Data. *J. Pharm. Sci.* **2013,** *102*, (9), 3239-3251.

38. Pearce, R. G.; Setzer, R. W.; Davis, J. L.; Wambaugh, J. F., Evaluation and calibration of high-throughput predictions of chemical distribution to tissues. *J. Pharmacokinet. Pharmacodyn.* **2017,** *44*, (6), 549-565.
